# Editorial: Perinatal infections among newborns in African countries: under-recognized, under-resourced and under-treated – a call for action

**DOI:** 10.3389/fpubh.2024.1439757

**Published:** 2024-06-21

**Authors:** Tinsae Alemayehu, Lisa Frigati

**Affiliations:** ^1^Department of Paediatrics and Adolescent Health, Faculty of Medicine, University of Botswana, Gaborone, Botswana; ^2^Department of Pediatrics and Child Health, St. Paul's Hospital Millennium Medical College, Addis Ababa, Ethiopia; ^3^Department of Paediatrics and Child Health, Stellenbosch University and Tygerberg Hospital, Cape Town, South Africa

**Keywords:** congenital infection, perinatal infection, Africa, children, congenital CMV (cCMV), perinatal tuberculosis, congenital syphilis, group B streptococcus (GBS)

This special edition features a collection of articles highlighting some of the burden and spectrum of perinatal infections in African countries and the challenges pediatricians face in diagnosing and treating them. The aim of this edition is to discuss the epidemiology and clinical presentations of perinatal infections in African countries, compare current diagnostic and treatment practice with existing practice in Africa and offer recommendations on the way forward. The Research Topic includes articles of varying topics: early-onset group B streptococcal disease, congenital syphilis, perinatal tuberculosis, and congenital cytomegalovirus, authored by researchers from diverse settings and regions of Africa. The edition does not address other important perinatal infections such as Zika, rubella, hepatitis B and C, HIV, varicella zoster, toxoplasmosis, malaria and herpes simplex virus.

In many African settings, perinatal infections are often not screened for, difficult to diagnose and difficult to treat. Surveillance for congenital anomalies that may be sequelae of *in-utero* infections are not routine. There are competing disease burdens and lack of access to appropriate treatments and logistic and implementation issues such as programs that work in silos. Access to amniocentesis, placental hisotology, Xrays, lumbar puncture, infant audiology testing, brain MRI are often limited and lead to suboptimal diagnosis and management of congenital infections, Global shortages of penicillin and lack of access to ganciclovir may also impact optimal prevention and treatment strategies.

Although not a “traditional” congenital infection, Streptococcus agalactiae has a significant impact on neonatal mortality (1). *Early-onset group B streptococcal disease in African countries and maternal vaccination strategies*, by Dangor et al.: this perspective article discusses invasive group B streptococcal (GBS) infection – a perinatal infection with twice higher burden and four times higher mortality in African countries as compared with the rest of the world (2, 3). This article reviews the existing gaps in knowledge of perinatal GBS epidemiology in Africa and narrates current prevention strategies including vaccine candidates. It also highlights opportunities and challenges in African countries to design pre- and post-vaccine comparison studies to identify changes in vaccine- and non-vaccine genotypes and disease trends.

There has been a global resurgence of syphilis. In 2020, 425 cases/100,000 live births were reported (4). An estimated 1 million pregnant women worldwide are diagnosed with syphilis annually, with the highest burden in sub-Saharan Africa and between 2012 and 2016, there were 1,120 cases per 100,000 livebirths in the African region between, compared with 19 cases per 100,000 livebirths in the European region (5).

*The prevalence of gestational syphilis in Malawi between 2014 and 2022: spatiotemporal modeling of population-level factors*, by Chirombo et al.: while most of the active gestational syphilis globally are diagnosed in resource-limited settings, < 10% of those are diagnosed and treated (6). An estimated 206,000 adverse pregnancy outcomes per year occur due to maternal syphilis in African countries (7). The authors of this original research aimed to identify high risk areas for gestational syphilis in Malawi. The findings of this study also help in following the prevalence trends in the study years and stratifying associated risks based on routine surveillance data and health surveys.

*Placental weights of neonates born with symptomatic congenital syphilis*, by Pillay et al.: previous studies have noted placentae in congenital syphilis weigh significantly more than in uninfected newborns due to effects of chronic inflammation (8). This brief report is a sub-study of previous research into the clinical presentation and outcomes of infants with symptomatic congenital syphilis. It describes a retrospective study from a tertiary hospital in South Africa, comparing the placental weights of neonates with symptomatic congenital syphilis with population based placental centiles. It also explains the differences in placental weight abnormality across gestational ages and birth weights of affected newborns.

*Perinatal tuberculosis – An approach to an under-recognized diagnosis*, by Schaaf et al.: infants born to mothers with tuberculosis (TB) have increased risk of acquiring the infection, developing active disease and progression to severe and disseminated diseases in their first year of life (9). There is a dearth of guidelines on the management of exposed infants (9). This mini-review article aims to increase an awareness of TB in young infants and provide guidance on antenatal and postnatal evaluation and management of newborns delivered to mothers with active TB. The paper also highlights research gaps in early diagnosis as well as pharmacokinetics of anti-tubercular drugs in newborns.

*Congenital cytomegalovirus in Sub-Saharan Africa - a narrative review with practice recommendations*, by Payne and Barnabas: congenital cytomegalovirus (cCMV) infection is one of the commonest causes of congenital and perinatal infections worldwide and is the commonest cause of sensorineural hearing loss in countries which have implemented routine childhood rubella vaccination programs (10). The disease burden is three-times higher in low- and middle-income countries while HIV-exposed newborns were noted to have a six-folds higher risk of cCMV infection (11, 12). This narrative review describes the epidemiology, presentation, principles of diagnosis and treatment of cCMV and identifies existing challenges in sub-Saharan Africa and potential solutions.

The articles in this Research Topic highlight current epidemiologic knowledge about congenital and perinatal infections in Africa, while underlining research gaps. They offer a summary of diagnostic and treatment recommendations but also describe the challenges faced in identifying affected children. This article collection calls for congenital and perinatal infections to get the attention they deserve as they affect hundreds of thousands of African children, the large majority being undiagnosed and thus, left unmanaged suffering long-term sequelae.

## Author contributions

TA: Conceptualization, Project administration, Resources, Supervision, Validation, Writing – original draft, Writing – review & editing. LF: Resources, Supervision, Validation, Writing – review & editing.
